# Perspectives of U.S. harm reduction advocates on persuasive message strategies

**DOI:** 10.1186/s12954-023-00849-z

**Published:** 2023-08-18

**Authors:** Sarah A. White, Rachel Lee, Alene Kennedy-Hendricks, Susan G. Sherman, Emma E. McGinty

**Affiliations:** 1 Health Policy and Management, Johns Hopkins Bloomberg School of Public Health, 624 N. Broadway St., Baltimore, MD 21205 USA; 2 Johns Hopkins University, 3400 N. Charles St., Baltimore, MD 21218 USA; 3 Health, Behavior and Society, Johns Hopkins Bloomberg School of Public Health, 624 N Broadway St., Baltimore, MD 21205 USA; 4 Division of Health Policy and Economics, Weill Cornell Medicine, 402 E. 67th St., New York, NY 10065 USA

**Keywords:** Harm reduction, Message strategies, Qualitative research

## Abstract

**Background:**

The messages used to communicate about harm reduction are critical in garnering public support for adoption of harm reduction interventions. Despite the demonstrated effectiveness of harm reduction interventions at reducing overdose deaths and disease transmission, the USA has been slow to adopt harm reduction to scale. Implementation of evidence-based interventions has been hindered by a historical framing of drug use as a moral failure and related stigmatizing attitudes among the public toward people who use drugs. Understanding how professional harm reduction advocates communicate to audiences about the benefits of harm reduction is a critical step to designing persuasive messaging strategies.

**Methods:**

We conducted qualitative interviews with a purposively recruited sample of U.S. professional harm reduction advocates (*N* = 15) to examine their perspectives on which types of messages are effective in persuading U.S. audiences on the value of harm reduction. Participants were professionals working in harm reduction advocacy at national- or state-level organizations promoting and/or implementing harm reduction. Semi-structured interviews were audio-recorded, transcribed, and analyzed using a hybrid inductive/deductive approach.

**Results:**

Interviewees agreed that messages about the scientific evidence demonstrating the effectiveness of harm reduction approaches are important but insufficient, on their own, to persuade audiences. Interviewees identified two overarching messaging strategies they perceived as persuasive: using messages about harm reduction that align with audience-specific values, for example centering the value of life or individual redemption; and positioning harm reduction as part of the comprehensive solution to current issues audiences are facing related to drug use and overdose in their community. Interviewees discussed tailoring messages strategies to four key audiences: policymakers; law enforcement; religious groups; and the family and friends of people who use, or have used, drugs. For example, advocates discussed framing messages to law enforcement from the perspective of public safety.

**Conclusions:**

Interviewees viewed messages as most persuasive when they align with audience values and audience-specific concerns related to drug use and overdose death. Future research should test effectiveness of tailored messaging strategies to audiences using experimental approaches.

**Supplementary Information:**

The online version contains supplementary material available at 10.1186/s12954-023-00849-z.

## Introduction

The magnitude of the United States’ (U.S.) overdose crisis has accelerated in recent years, with drug overdose deaths increasing nearly 50% since 2019 to over 105,000 in 2022 [1, 2]. Contributing to this acceleration of overdose death is an increasingly adulterated drug supply including illicitly-manufactured-fentanyl and xylazine [3]. As part of their response, the U.S. federal government has incorporated harm reduction in its Overdose Prevention Strategy for the first time [4]. This national strategy includes grant funding for syringe service programs and naloxone distribution, federal funding allowances for fentanyl test strips, and support for research networks focused on harm reduction. Despite harm reduction’s demonstrated effectiveness at reducing overdose deaths [5–8], the USA has been slow to adopt harm reduction interventions on a wide scale in many locations in need. Slow adoption has been driven by interrelated factors including a historical framing in the U.S. of drug use as a moral failing, low public support for harm reduction interventions, and the corresponding preference for interventions aimed at abstinence from drug use [9–14]; additionally, stigmatizing attitudes toward people who use drugs and racist misperceptions that drug use is concentrated among minority populations have negatively impacted the adoption of harm reduction in the U.S. [1, 6, 15].

Messages used to communicate about harm reduction are critical in garnering public support for adoption of harm reduction approaches in the U.S. Previous studies have demonstrated that messaging strategies can increase public support for particular harm reduction interventions, like naloxone distribution and safe consumption sites, through factual messages about harm reduction and sympathetic narratives humanizing people who use drugs [17, 18]. Another study found that messages re-framing “safe consumption sites,” where people can legally use pre-obtained drugs under medical supervision, as “overdose prevention sites” increased audience support for legalizing these sites [10]. However, a study conducted in 2017 showed that U.S. adults found arguments against harm reduction services (e.g., that they enable drug use) more persuasive than arguments supporting their implementation [1, 3].

Understanding professional harm reduction advocates’ persuasive communication experiences can illuminate lessons learned. Previous studies have summarized perspectives of advocates from the United Kingdom and Australia on effective communication strategies in harm reduction debates [19], but there are no similar studies highlighting advocates’ perceptions of effective communication strategies in the U.S. This qualitative study fills this gap by interviewing harm reduction advocates about their perspectives on effective messaging strategies for persuading U.S. audiences about the value of harm reduction approaches in addressing overdose deaths.

## Methods

We interviewed 15 professional harm reduction advocates with experience advocating for harm reduction approaches at the national, state, and/or local levels in the U.S. Participants were purposively selected for recruitment if they had a professional role involving harm reduction advocacy at leading national- or state-level organizations focused (heavily, if not solely) on implementation of harm reduction policies and interventions in the U.S. We also used snowball sampling by asking participants to identify additional individuals with relevant advocacy expertise for potential recruitment. We recruited a geographically diverse sample of advocates to ensure discussions encompassed different U.S. regions and levels of urbanicity/rurality. Interviews were conducted until data saturation was reached and no new themes emerged from interviewees related to perceived persuasive messaging strategies.

A semi-structured interview guide was created based on existing literature and our specific research goal of understanding advocates’ perceptions of persuasive harm reduction communication strategies in the U.S. Additionally, the research questions were informed by the study team’s previous experience conducting qualitative interviews on harm reduction programs and analyzing message strategies on support for drug policy. The interview guide (see Additional File 1) began with grand tour questions about interviewees’ professional experience in harm reduction advocacy [20–23]. Following this, research-driven questions focused on two domains: (1) most persuadable/least persuadable audiences; and (2) persuasive messaging strategies to convincing the general U.S. public on harm reduction.

Semi-structured interviews were conducted by a single study team member from February through May 2022. Participants were recruited via email. All interviews were conducted over the phone with an average duration of 47 min (ranging: 21–71 min). Interviewees were provided a $50 honorarium for their participation, through their choice of a gift card or pre-loaded debit card. An oral consent process was completed at the beginning of each interview. Following each interview, the study team member conducting the interviews wrote a brief memo summarizing major themes. Interviews were audio-recorded and transcribed for analysis.

For our analysis, we used an interpretivist epistemology focused on understanding harm reduction advocates’ subjective perspectives on persuasive communication strategies [24] .

Interview transcripts were analyzed using a hybrid inductive/deductive thematic analysis approach. The development of an initial codebook was informed by previous literature and the summaries created after each interview. The codebook was developed iteratively and piloted by two members of the study team through blind and independent double coding until the themes developed were consistent across reviewers. Coding and identification of themes and sub-themes were completed using NVivo. This research was reviewed and approved by the Johns Hopkins Bloomberg School of Public Health Institutional Review Board.

## Results

Interviewees included eight females and seven males, all with professional roles advocating for harm reduction policies (Table 1). Eight interviewees were advocates in harm reduction-focused agencies and coalitions, including two members of a coalition of people who use drugs and/or have been impacted by the war on drugs, and seven interviewees were advocates in organizations conducting advocacy around harm reduction in addition to other issues (e.g., an organization focused on drug policy or viral hepatitis). Interviewees reported professional experiences including harm reduction advocacy directed to policymakers at the federal, state, and local levels (e.g., legislative testimony); advocacy activities directed to the public (e.g., opinion editorials and social media campaigns); and community-focused advocacy efforts (e.g., advocacy at a town hall meeting in support of a community’s proposed adoption of a harm reduction program). Multiple interviewees played dual roles as advocates and direct harm reduction service providers.

**Table 1:** Harm reduction experience among interviewees

Interviewee characteristics	# of interviewees (*n* = 15)
*Current organizational affiliations*	
Harm reduction agencies and coalitions	8
Organizations conducting advocacy on harm reduction and other issues	7
*Reported advocacy experiences*	
Advocacy to policymakers (e.g., legislative testimony)	14
Advocacy to the public (e.g., opinion editorials and social media campaigns)	12
Advocacy directed to specific communities (e.g., communities considering implementing a harm reduction program)	10
*Service provision*	
Harm reduction service provider (e.g., at drop-in centers or syringe services programs)	11

Interviewees agreed that messages about the scientific evidence demonstrating the effectiveness of harm reduction approaches are important but inadequate, on their own, to persuade U.S. audiences on the value of harm reduction. Interviewees identified two overarching messaging strategies they perceived as persuasive: using messages about harm reduction that align with audience-specific values; and positioning harm reduction as part of the comprehensive solution to current issues audiences are facing related to drug use and overdose in their community. Interviewees stressed that these values and issues vary by audience and that messages need to be tailored accordingly:

“One messaging system is not going to work with certain types of folks, but another messaging system will work with-- say, from my own local organizing, like in the city limits, very Democratic, Black-led. The organizing there is night and day from outside of [City Area Code]. Our perimeter is really two different messaging systems. I mean, that's the truth. And what works inside of [City Area Code] doesn't really work outside of it and vice versa. And maybe hopefully one day it will. But as a realistic kind of like, okay, ‘what's really going to work to get this law passed,’ you have to be like, ‘okay, do what you need to do. Talk in the way you need to talk. Dress in the way you need to dress.’ But on the inside of [City Area Code], where it's Black-led, I mean, that's where [State]’s harm reduction started.”

### Aligning messages on harm reduction with audience values

Four groups were consistently highlighted across interviewees as key audiences to tailor messaging strategies toward: policymakers, law enforcement, religious groups, and the family and friends of people who use, or have used, drugs. For policymakers, interviewees noted that arguments on the cost-effectiveness of harm reduction interventions, or a “value-for-money” framing, were sometimes useful. However, interviewees also reported that these messages should be paired with meaningful stories from constituents and messages emphasizing harm reduction’s role in promoting their community’s goals:

“So for policymakers, I think that efficacy in terms of reduction of infection, not encouraging drug use, all of the standard talking points about why harm reduction is good policy. Cost savings compared to the cost of HIV infections averted, those kinds of things. Very useful. That's one audience. I think that those arguments are pretty well developed. And I can think of a lot of papers that make the case, but, that that has not been incredibly compelling to, for example, even policymakers who might be charged with health allocation but don't want the moral risk of seeming to support something that goes against community values.”

For law enforcement, interviewees recommended framing harm reduction as a set of interventions that contribute to public safety, including the safety of both communities and officers. Interviewees’ examples included messages about how overdose prevention centers can decrease public drug use and its impact on officers and communities, and messages about how syringe service programs can decrease officers’ (and communities’) contact with used syringes:

“We're doing work with law enforcement to implement a needle-stick prevention law that was passed in the state several years ago… So, the law that said that if somebody discloses they have a syringe prior to being searched, then that syringe is exempted from charge -- at least they can't incur a paraphernalia charge from it… The side effect of that, though, is that if somebody is going to ask, "Do you have a syringe on you?" and then they actually don't charge the person for it after discovering it, you build some bridges in some unlikely places and you build support in unlikely places… So, the cop doesn't have to like the person that has syringes in their pocket. But they also don't need to go jamming their fingers into their pockets without asking. There's something that they gain from the behavior, whether they feel good about it or not.”

Notably, interviewees recognized concerns among harm reduction advocates, organizers, and service delivery providers about whether and how to involve law enforcement in advocacy and implementation efforts:

“So, it's like the harm reduction movement is splintered because some people feel like it's good to work with law enforcement and some people feel like law enforcement has only ever harmed communities of people who use drugs or engage in sex work.”

While these tensions were recognized, interviewees mostly agreed that engaging with law enforcement about harm reduction program implementation was critical because advocacy efforts to policymakers and local communities would often fail without law enforcement support:

“You can have everybody on the same page. But if prosecutor or somebody from law enforcement community walks in and says, ‘Our communities will be less safe because of this,’ everybody falls in line with that. Literally. ‘This will make us less safe,’ and everybody’s like, ‘Well, we’d love to talk about it, but it can’t be less safe.’”

For religious groups, interviewees recommended tailoring messages toward the compassionate values of that religion. Interviewees primarily discussed strategies for U.S. Christian audiences and framing harm reduction as aligning with Christian values around redemption. Interviewees highlighted international examples of faith leaders championing harm reduction programs, as well as domestic examples like Project Lazarus and GLIDE [25, 26]. However, interviewees also recognized that harm reduction efforts have historically been less accepted by U.S. individuals who identify as religious conservatives. Advocates expressed a particular need for individuals trusted by this audience, like religious leaders, to deliver authentic messages that resonate with their values:

“This is not messages that we employ as an organization, but it is messages that it's like, we will work with allies on the ground who come from those communities, or are part of those constituencies that then put it into their own words to kind of appeal to people's more compassionate nature through their church. Or through their faith. In terms of just what would you-- how would you want this person to be treated, right? As opposed to really a lot of talk about the interventions themselves, it's really about how would you want this person to be treated? What would it look like to treat this person with dignity and compassion?”

For family and friends of people who use drugs, interviewees reported framing harm reduction as an approach that values the life of their loved one:

“Family members definitely need their pain and challenges acknowledged. And their fears acknowledged. I think a lot of family members have a lot of fears. And so really grounding how you talk about harm reduction in terms of these are the people who will stay connected with them. These are the people who won't leave your loved one behind because they dropped out of treatment, right? I feel like so many family members want to get their loved one into treatment because that seems like the safest place for their loved ones to go. And then what happens is, oftentimes, their loved one is one of the 60% of people who started a treatment episode and didn't finish it. Then their loved one is left floating. So, if you want to talk about harm reduction with loved ones and even maybe with community members, it's this idea that it's still a touchpoint. It's still a place where they're going to get their needs met. They're going to be connected with someone, someone's keeping an eye on them, giving them connections to other resources.”

Interviewees noted that they often encounter, and must attempt to counter, oppositional messages that drug use devalues a person’s life. These oppositional messages may have previously resonated with the family and friends of people who use drugs; so, an additional strategy noted by interviewees as helpful for loved ones was utilizing stories of “changed minds”—peers in similar family situations that changed their minds about the value that harm reduction could provide to their loved one and their family:

“I do think the personal stories can also be useful there, particularly from family members or parents who had a change of heart. Who said ‘yeah, I did tough love for like 10 years and it didn't change a thing;’ ‘I ostracized the person from my family. I wouldn't let them come to Christmas. I tried to force them into treatment, and none of it worked. Ultimately what's most important to me now is that my son, or daughter, or brother, or sister, or uncle, or whoever is safe and is in a position if and when they're ready to try to abstain or to enter long-term recovery but they're able to do that and that harm reduction provides them this opportunity. It provides them opportunity for eventual recovery if that's what they want.’”

While interviewees emphasized this message in the context of family and friends of people who use drugs, they also noted that messages emphasizing the value of life were persuasive across many audiences:

“Preventing deaths, saving lives has to be the North Star. Harm reduction does that. We have to keep-- number one, first and foremost above everything else, is keeping people alive. Harm reduction does that more effectively than anything else we have.”

### Framing harm reduction as part of comprehensive solution to audience concerns

Another communication strategy interviewees perceived as persuasive was positioning harm reduction as part of the comprehensive solution needed to address audiences’ specific concerns related to drug use and overdose in their community. Interviewees noted that audiences’ concerns were often influenced by personal and anecdotal experiences, and that persuasive messaging strategies addressed the concerns raised by those experiences. For example, the advocates interviewed reported using messages emphasizing how harm reduction interventions like naloxone distribution can decrease overdose deaths in communities that are heavily impacted by rising deaths. When audience concerns focused on enabling drug use, interviewees described contextualizing how harm reduction does not encourage new users and offers needed, immediate, and integrated support to people in their community. As a cautionary note, many interviewees reported hearing audience concerns about community-level harms that they had data to refute; however, they stressed that arguing on technicalities is not persuasive and noted that they work to avoid dismissing an individual’s concerns.

When communicating about harm reduction with representatives of organizations in the community, for example health systems or major employers within a community, the advocates interviewed suggested tailoring messages to emphasize how harm reduction can address specific burdens faced by that organization:

“Highlighting how harm reduction will benefit the institution or organization I'm talking to has also been very, very good for messaging. So, explaining that by decreasing HIV, we're going to save X amount of dollars financially at a county level. Right? That's a big benefit there. If we tell a sheriff that you're not going to have people puking and dying in your jail if people get the medication [for opioid use disorder], that's a huge pain point for them and a benefit for them. So, tying the harm reduction interventions to why that's beneficial to somebody else is always good for messaging… Law enforcement, specifically the medical community, specifically legislators, policymakers, municipality leaders, they need to see an overall picture of the cost of SUD on their workforce, on their emergency departments, on their jails, and they need to be highlighted how the cost savings related to syringe service predications, medications, naloxone, how it will help the cost in their emergency departments, how it will reduce recidivism in their jails, all these public health-- the way these public health interventions will help.”

When discussing persuasive messaging strategies for communities considering harm reduction interventions, interviewees reported using messages that highlight the community-level wellness that harm reduction can promote. Additionally, messages need to recognize the harms from drug use, overdose, and the criminalization of drug use that the community has experienced. For some audiences, interviewees posited that harm reduction should be framed as improving community wellness by addressing harms like overdose deaths and syringe litter:

“I mean when I'm talking about the people who are afraid, those folks think you’re going to take their property values down; or you have parents who have lost kids, and, well, you come into their community, and they see you as killing more kids. Part of the [harm reduction] campaign, really, is actually listening to what they have to say and being smart enough to be able to respond to what they have to say. Which I don’t think the feds are in a position to do yet—they don’t have the language. So, there's the gentle piece of it where you talk about the life-saving things, you talk about the amount of people who enter drug treatment, you talk about how many people got cured of hepatitis C, those kind of things.”

For audiences from minority communities, particularly Black communities, interviewees noted that it was critical to frame harm reduction as alleviating community-level harms from the historic and continued discriminatory criminalization of drug use in the U.S:

“Many poor, historically African American neighborhoods in [Interviewee’s community] would be opposed to an overdose prevention center. Why is that? Is it because their other needs aren't being met? Because they feel ignored? Because they feel discriminated against? Because they feel they've been historically criminalized and stigmatized for substance use and incarcerated? Depicted in the media as less-than-human? Or all of these other things, right? - Not worthy of treatment, worthy of prison, jail? … I always say we need to go back to the 1980s, 1990s crack epidemic, so to speak, because people, in their communities, have lived experiences and have been historically and structurally traumatized by our collective responses to substance use. And a lot of people that carry those [stigmatizing] beliefs have come from communities that may have been harmed.”

## Discussion

This is the first study focused on describing harm reduction advocates’ perceptions of effective message strategies for persuading U.S. audiences on the value of harm reduction approaches. Harm reduction advocates stressed the need to develop message strategies tailored to intended audiences. Advocates viewed messages that align with audiences’ values and messages that position harm reduction as part of the comprehensive solution to issues specific audiences are facing as persuasive. The perspectives of the U.S. harm reduction advocates interviewed in this study were consistent with views of advocates in the United Kingdom (U.K.) and Australia [19] who reported that “value-neutral” message strategies were not effective in political debates on harm reduction. There are discussions among harm advocates about how to balance evidence-based, public health-focused messaging strategies with values-based, or morally focused, messages [19, 27–31]. Some are in favor of emphasizing evidence demonstrating the effectiveness of harm reduction interventions to circumvent moral concerns around drug use [29]. Others argue that values shape deliberations of drug policy so, in practice, moral concerns are impossible to ignore [31]. Analyses of policymaker debates and media reports of harm reduction and drug policies in Sweden, Australia, and the U.K. show that evidence played a minor role in harm reduction and drug policy debates. Instead, debates often focused on competing values around drug use—i.e., that it is a moral failing versus the consequence of either an unjust society or a disease [19, 27, 31, 32]. Interviewees in our study recognized that evidence-focused arguments were not enough to convince skeptical policymakers but viewed being ready to use these messaging strategies when questioned about the evidence was critical.

Learning from advocates about their experiences engaging a range of audiences on the value of harm reduction is a helpful first step in developing harm reduction messaging strategies for widespread use, e.g., through public communication campaigns. However, it is critical to use rigorous experimental methods to evaluate the effectiveness of messages aimed at increasing support for harm reduction policies and programs. Messaging strategies that are widely deployed without rigorous testing can result in costly dissemination with unintended effects. For example, a national campaign aimed at reducing public stigma by framing mental illness as being on a par with other chronic diseases, such as diabetes, has been shown, by some measures, to have increased stigma toward people with mental illness [33]. To ensure that values-based messages on harm reduction do not lead to unintended effects on stigma and policy support—since messages beneficial for one can have no effect or be detrimental to the other [34–36]—future message strategies need to be evaluated before widescale dissemination.

While evidence-based messaging strategies are needed to further increase support for and uptake of harm reduction interventions, harm reduction is gaining traction in the U.S. For example, the federal Overdose Prevention Strategy includes grant funding for naloxone syringe service programs, New York City has two overdose prevention centers operated by OnPoint, and additional underground overdose prevention centers operate in the U.S [35, 37, 38]. But, critical gaps remain. For example, 13 states have laws that ban syringe service programs from operating, and 44 states’ drug paraphernalia laws, which criminalize the possession and/or sale of illicit drug paraphernalia, include fentanyl test strips in their definitions [39]. Persuasive communication strategies are a key component to increase support for scaling evidence-based harm reduction interventions in the U.S.

## Limitations

This study should be considered in the context of several limitations. First, we summarize the perspectives of a small number of U.S. advocates with professional experience advocating for harm reduction interventions and policies. Second, none of the messages described in this manuscript have undergone the experimental testing we recommend in our discussion. Our study highlights messaging strategies that our interviewees have found to be persuasive, but these strategies have not been empirically evaluated. Third, our findings may not generalize to non-U.S. audiences, whose perceptions of persuasive message strategies likely differ from those in the U.S.

## Conclusion

A sample of professional advocates with experience advocating for harm reduction approaches at the national, state, and/or local levels in the U.S. viewed message strategies as persuasive when they align with audience-specific values and position harm reduction as part of the comprehensive solution to issues audiences are facing around drug use and overdose deaths. Further evaluation of tailored message strategies for audiences is critical to understand their effectiveness in garnering audience support for harm reduction approaches.

### Supplementary Information

**Additional file 1:** Semi-structured interview guide used for qualitative interviews with harm reduction advocates.

## Data Availability

The qualitative datasets generated during the current study are not publicly available due to confidentiality of participants but are limitedly available from the corresponding author on reasonable request.

## Abbreviations

U.K.: United Kingdom
U.S.: United States of America
