# Supplementary material for: Perspectives of U.S. harm reduction advocates on persuasive message strategies
Source: Harm Reduct J. 2023 Aug 18;20:112. doi: 10.1186/s12954-023-00849-z (PMC10436451; doi:10.1186/s12954-023-00849-z)
Supplement: Supplementary file 1 — Additional file 1: Semi-structured interview guide used for qualitative interviews with harm reduction advocates. [file 12954_2023_849_MOESM1_ESM.docx]

**Appendix A. Interview Guide for Harm Reduction Advocates**

***Grand-tour questions:***

1. Tell me about your professional role and your general experience with harm reduction messaging.
2. How do you define harm reduction?
3. Do you have any experience developing and delivering messages designed to increase support for harm reduction policies and programs? This could be formal communication campaigns disseminated in a community or through more informal community conversations, advocacy work, etc.?
   1. *Prompt if yes: what strategies have you used to convince people about the value of harm reduction?*
   2. *Prompt: who was most receptive to your messages?*
   3. *Prompt: who was least receptive to your messages?*

***Key barriers/facilitators:***

1. What are the key barriers we must overcome to convince the general public about the value of harm reduction?
2. What are the key facilitators to convincing the general public about the value of harm reduction?

***Persuasive messages:***

1. Do we need to tailor harm reduction messages for specific groups? Tell me more.
   1. *Prompt: are there specific groups that you think are more/less open to harm reduction messaging?*
   2. *Prompt: are there specific groups that you think are good targets for persuasive messaging, in that their views can be changed? Are there groups who you think are not good targets, because their views cannot be changed?*
2. What types of messages do you think are most useful at improving audiences’ understanding of what harm reduction is and how it works?
   1. *Prompt: what types of messages are most useful at improving* ***[Specific group mentioned in Q6]*** *audiences’ understanding of harm reduction?*
3. What types of messages do you think are most useful at improving audiences’ perceptions of the effectiveness of harm reduction approaches in achieving public health goals, like preventing drug overdose?
   1. *Prompt: what types of messages are most useful at improving* ***[Specific group mentioned in Q6]*** *audiences’ perceptions of the effectiveness of harm reduction approaches in achieving public health goals?*
4. What types of messages do you think are most useful at improving audiences’ support for implementing harm reduction approaches?
   1. *Prompt: what types of messages are most useful at improving* ***[Specific group mentioned in Q6]*** *audiences’ support for implementing harm reduction approaches?*
5. Do you think that different message strategies are needed for different harm reduction interventions? Tell me more. (e.g. syringe services, overdose prevention sites, naloxone distribution and training)
   1. *Prompt: are there specific harm reduction interventions that you think general audiences are more/less open to?*

***Ineffective messages:***

1. Are there messages intended to increase audiences’ understanding of and support for harm reduction that you think do not work as intended? Tell me more.
2. Are there messages intended to increase audiences’ understanding of and support for harm reduction that you think are actually counterproductive for some or all audiences? Tell me more.

***Oppositional messages:***

1. What messages opposing harm reduction do you think audiences find most persuasive?
2. What do you think are the best strategies for combatting these messages?
